# Epidemiology of foodborne diseases caused by *Salmonella* in Zhejiang Province, China, between 2010 and 2021

**DOI:** 10.3389/fpubh.2023.1127925

**Published:** 2023-02-01

**Authors:** Yue He, Jikai Wang, Ronghua Zhang, Lili Chen, Hexiang Zhang, Xiaojuan Qi, Jiang Chen

**Affiliations:** Department of Nutrition and Food Safety, Zhejiang Provincial Center for Disease Control and Prevention, Hangzhou, China

**Keywords:** epidemiology, public health, foodborne disease (FBD), surveillance system, *Salmonella*

## Abstract

**Objective:**

*Salmonella* infection is a common cause of bacterial foodborne diseases (FBDs) globally. In this study, we aimed to explore the epidemiological and etiological characteristics of *Salmonella* infection from 2012–2021 in Zhejiang Province, China.

**Methods:**

Descriptive statistical methods were used to analyze the data reported by the Centers for Disease Control and Prevention at all levels in Zhejiang Province through the China National Foodborne Diseases Surveillance Network from 2012–2021.

**Results:**

A total of 11,269 *Salmonella* cases were reported, with an average positive rate of 3.65%, including 1,614 hospitalizations. A significant seasonal trend was observed for *Salmonella* cases, with the highest rate over the summer period, peaking from May to October, accounting for 77.96%. The results indicated a higher positive rate among respondents aged 0–4 years, especially for the scattered children (*P* < 0.05). The highest number of *Salmonella* infections were caused due to contaminated fruit and fruit products. Households (54.69%) had the most common exposure settings. Serotypes analysis revealed that *Salmonella typhimurium* (36.07%), *Salmonella enteritidis* (15.17%), and *Salmonella london* (6.05%) were the dominant strains among the 173 serotypes. Diarrhea, abdominal pain, fever, nausea, and vomiting were the main symptoms of these serotypes.

**Conclusions:**

FBDs caused by *Salmonella* are important issues for public health in Zhejiang Province, and there is a need to focus on the epidemiological and etiological characteristics to control *Salmonella* infections.

## 1. Introduction

Foodborne diseases (FBDs) represent global public health issues that result in considerable morbidity and mortality in all age groups and are a hurdle to socioeconomic development. The World Health Organization (WHO) estimated that there were ~600 million (almost 1 in 10) cases caused by contaminated food, resulting in 33 million disability-adjusted life years (DALYs) in 2010 (1, 2). Generally, FBDs occur due to specific pathogens, such as the bacteria, viruses, parasites, fungi, and mycotoxins, and prions, environmental factors like contamination during the production, processing, transport, and storage phases, as well as the conditions of the host's immune system (3, 4). The most frequent causes of FBDs worldwide are bacterial pathogens, the most important being *Salmonella, Vibrio parahaemolyticus, Listeria monocytogenes, Staphylococcus aureus*, and some other pathogens (4–8).

*Salmonella*, a vital microorganism responsible for FBDs, mainly exists in foods of animal origin, especially raw poultry, raw meat, eggs, and their products (9, 10). Annually, *Salmonella* causes ~200 million to over 1 billion infections worldwide, with 93 million cases of gastroenteritis and 155,000 deaths, and 85% of illnesses which are food-linked (11–13). Due to cross-contamination in the production process, the bacteria may get transferred onto these products (14). Meat and poultry products were a good environment for the growth of *Salmonella* because of rich content of nutrients and water (15). Besides, fruit and vegetable products contaminated by animal fecal flora could act as a breeding ground for *Salmonella* (16). Moreoover, in 2017, 10,000 cases of *Salmonella* infections were registered in Poland, and the incidence rate per 100 thousand population was 26.0% (17). Human challenge studies have demonstrated that patients can develop food poisoning after ingesting *Salmonella*, which has influenza-like symptoms including nausea, vomiting, abdominal pain, and diarrhea (18). At present, *Salmonella* has more than 2,500 serotypes worldwide, and more than 200 serotypes have been identified in China. During 1996–2014, *S. typhimurium, S. enteritidis*, and *S. newport* were the three most common serotypes reported by the Foodborne Diseases Active Surveillance Network (FoodNet) sites of the Centers for Diseases Control and Prevention (CDC) in the US (19).

FBDs surveillance aims to monitor food contamination and harmful factors, and reduce the burden of illness due to contaminated food. There are several different types of FBD surveillance systems, including event-based surveillance, indicator-based surveillance, and integrated food chain surveillance (20). Since 2011, China has successively established a web based FBD surveillance platform, which includes the Foodborne Disease Outbreaks Surveillance System (FDOSS), Foodborne Disease Surveillance and Reporting System (FDSRS), National Molecular Traceability Network for Foodborne Diseases (TraNet), and other surveillance systems (21). The FDSRS system is applied for collecting information about foodborne disease patients visiting medical institutions at all levels, including self-reported suspicious food exposure and pathogen detection results. Zhejiang Province, on the southeast coast of China, is located at 27°02'N to 31°11'N and 118°01'E to 123°10'E, has 35,100 health institutions (including village clinics), with 1,486 hospitals and 103 CDCs (22). In this study, the national FBDs surveillance data collected over 10 years were used to describe the epidemiological characteristics, food vehicles, and setting distribution of foodborne gastroenteritis caused due to *Salmonella* infections in Zhejiang Province.

## 2. Methods

### 2.1. Diagnostic criteria for *Salmonella* FBDs

The diagnostic criteria were mainly based on the clinical symptoms and microbiological evidence. Suspected cases were considered to have an acute gastrointestinal illness (AGI) if they met one or both of the following clinical symptoms: (1) diarrhea, defined as three or more loose stools within 24 h, accompanied by abnormal fecal characteristics, and (2) vomiting (accompanied by content). Microbiological evidence was obtained when *Salmonella* was isolated from suspected food items, equipment, utensils, or when a simultaneous serotype of *Salmonella* was detected in the vomit or feces of multiple patients.

### 2.2. Data collection

The Zhejiang Provincial CDC (ZJCDC) has been collecting FBD- relevant data through the China National Foodborne Diseases Surveillance Network (NFDSN) since 2012. One hundred and one hospitals were asked to detect *Salmonella* pathogens and their corresponding subtypes for all suspected foodborne disease cases, and reported illnesses through NFDSN since 2012. In this study, cases reported by 101 hospitals in Zhejiang Province between 2012 and 2021 were included. Epidemiologists from the health departments first conducted an investigation to ascertain the full extent of the foodborne illness, and the information collected for each case includes the reporting region, date of occurrence, setting, etiology, food categories, number of illnesses/hospitalizations, and other details. Unknown etiology refers to foodborne disease cases in which the confirmed etiology has not been identified. Settings were classified into eight categories. Food items were identified as sources of disease through epidemiological or laboratory methods and were classified into 14 categories. Food that could not be determined was classified as “Unknown.” The GIS map data of Zhejiang Province was downloaded from the national basic geographic information center of China (http://bzdt.ch.mnr.gov.cn/).

### 2.3. Standard laboratory protocol for *Salmonella*

Fresh stool specimens or anal swabs were collected from cases. The best specimens were a fecal specimen, anal swab was used only when the patient had no stool specimen. Specimens collected were tested as soon as possible. Specimens placed in the culture-Blair medium were tested within 24 h of refrigeration. Fresh fecal samples were placed in clean, dry containers without soap or disinfectant residue, and sent for examination within 8 h of refrigeration.

Isolation and identification of *Salmonella* were performed as described in the Operation Procedure for *Salmonella* Inspection in the Foodborne Disease Surveillance Work Manual of the National Center for Food Safety Risk Assessment. In brief, the above specimens were placed in SBG augmenting solution and cultured at 36°C for 18 to 24 h. Furthermore, after gently shaking the expanding liquid tube we applied 1 ring line to the *Salmonella* chromogenic medium or XLD AGAR plate and incubated it at 36 ± 1°C for 18 to 24 h. We picked three to five suspected colonies, inoculated in TSI AGAR, lysine decarboxylase, and nutrient AGAR plates, at 36 ± 1°C for 18 to 24 h. A single colony was scraped from a nutrient AGAR plate for systematic biochemical identification. Either of biochemical identification kit or automatic microbial biochemical identification system can be selected for identification.

The *Salmonella* serovar was identified with specific O and H antiserum samples according to the Kauffmann–White scheme as described in the instructions provided by the manufacturer of the antiserum samples (Statens Serum Institute, SSI).

### 2.4. Data analysis

All the data was analyzed using IBM SPSS Statistics for Windows, version 22.0 (IBM Crop., Armonk, NY, USA). Open-source software QGIS (Quantum GIS version 3.22.9) was used to map the spatial distribution of cases with positive detection rates caused by *Salmonella* between 2012 and 2021.

The total positive detection and hospitalization rates were calculated for *Salmonella*, and a linear trend test was used to detect the change in the positive detection and hospitalization rates annually. Chi-square tests were used to compare the relationship between demographic characteristics and the positive rate, including sex, age, annual distribution, season, and area. Fisher's exact test was used if the conditions were not met in the chi-square test. A *post-hoc* test was used for pairwise comparisons. The seasons were classified as winter (December to February), spring (March to May), summer (June to August), and autumn (September to November). *P* < 0.05 was considered as significant.

## 3. Results

### 3.1. General epidemiological characteristics

**Figure 1:**
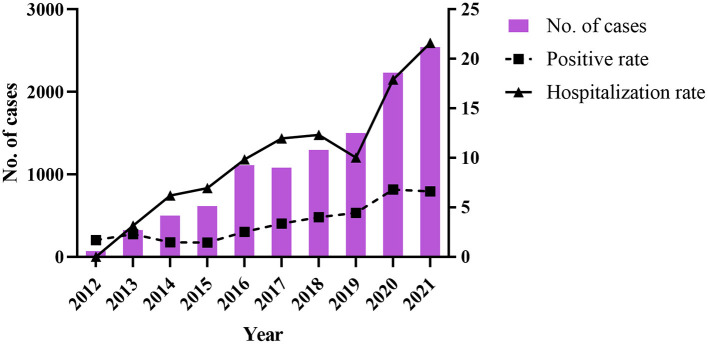
The change of number of cases, positive rate, and hospitalization rate of *Salmonella* during 2012–2021.

Between 2012 and 2021, 420,736 suspected FBD cases were reported in medical institutions at all levels in 11 cities in Zhejiang Province, and 308,326 stool samples were collected for *Salmonella* testing. The total positive rate was 3.65% (11,269/3,08,326). The positive detection rate for *Salmonella* increased from 1.69 to 6.61% during 2012–2021 (Table 1, Figure 1), and the number of reported confirmed cases increased, especially in 2020 (6.80%) and 2021 (6.61%) (Table 1). A significant increase in the hospitalization rate was observed during the study period (Table 1, Figure 1).

**Table 1:** Demographic characteristics and *Salmonella* positive rate in Zhejiang Province from 2012 to 2021.

**Variable**	**Cases**		**Hospitalizations^a^**		**Positive rate (%)**	**χ^2^**	***P***
	***n***	**%**	***n***	**%**			
**Annual distribution**						0.103	<0.001
2012	69	0.61	0	0.00	1.69		
2013	324	2.88	51	3.16	2.30		
2014	502	4.45	100	6.20	1.46		
2015	615	5.46	112	6.94	1.44		
2016	1,109	9.84	159	9.85	2.54		
2017	1,078	9.57	193	11.96	3.36		
2018	1,296	11.50	199	12.33	4.01		
2019	1,502	13.33	162	10.04	4.45		
2020	2,234	19.82	289	17.91	6.80		
2021	2,540	22.54	349	21.62	6.61		
**Area**						0.065	<0.001
Hangzhou	1,306	11.59	40	2.48	2.78		
Ningbo	1,181	10.48	150	9.29	3.74		
Wenzhou	1,050	9.32	108	6.69	2.48		
Jiaxing	788	6.99	77	4.77	3.35		
Huzhou	357	3.17	94	5.82	1.68		
Shaoxing	899	7.98	136	8.43	4.29		
Jinhua	1,252	11.11	278	17.22	3.80		
Quzhou	820	7.28	261	16.17	4.54		
Zhoushan	536	4.76	85	5.27	3.57		
Taizhou	1,907	16.92	157	9.73	6.57		
Lishui	1,173	10.41	228	14.13	4.39		
**Season**						0.079	<0.001
Spring	2,052	18.21	310	19.21	3.32		
Summer	5,660	50.23	799	49.50	5.21		
Autumn	3,094	27.46	432	26.77	3.66		
Winter	463	4.11	73	4.52	0.87		
**Sex**						6.275	0.012
Male	6,155	54.62	928	57.50	3.74		
Female	5,114	45.38	686	42.50	3.57		
**Age (year)**						0.005	<0.001
0–4	4,060	36.02	777	48.14	8.79		
5–14	607	5.39	109	6.75	3.25		
15–24	729	6.47	42	2.60	1.80		
25–44	1,999	17.74	84	5.20	2.03		
45–59	1,765	15.66	155	9.60	3.22		
≥60	2,109	18.72	447	27.70	4.34		
**Occupation**						0.007	<0.001
Farmer	3,008	26.69	435	26.95	3.44		
Scattered kids	3,136	27.83	609	37.73	13.02		
Worker	684	6.07	40	2.48	3.08		
Student	701	6.22	77	4.77	2.39		
Official staff	408	3.62	18	1.12	1.62		
Unemployed	560	4.97	66	4.09	2.53		
Kids in kindergarten	1,106	9.81	223	13.82	4.47		
Retirees	367	3.26	73	4.52	3.01		
Others	1,089	9.66	58	3.59	1.98		
Unknown	210	1.86	15	0.93	2.09		

a Hospitalization of cases with positive detection results.

**Figure 2:**
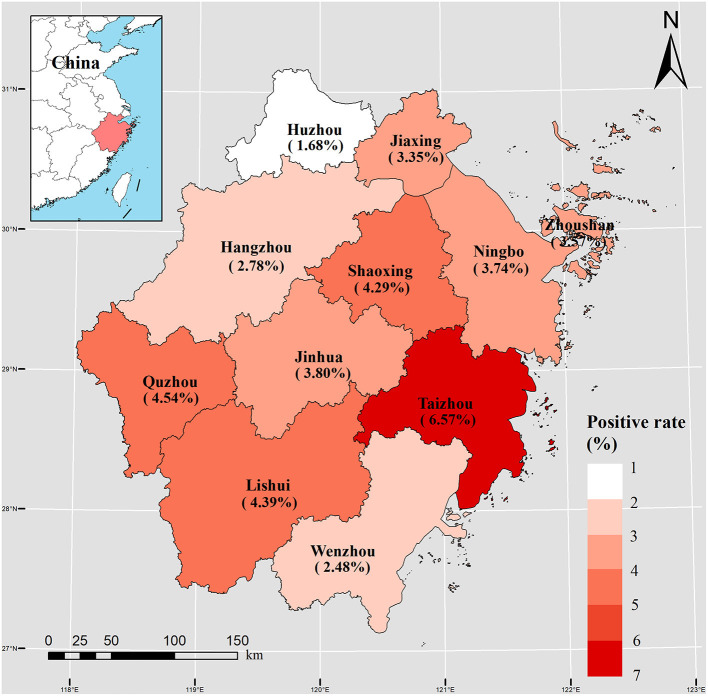
Spatial distribution of 11,269 *Salmonella* cases during 2012–2021 in 11 cities of Zhejiang Province.

The regional distribution of cases with positive *Salmonella* infection among 11 cities is shown in Figure 2. City of Taizhou, Quzhou, and Lishui cities had a positive rate of 6.57% (1,907 cases), 4.54% (820 cases), and 4.39% (1,173 cases), respectively (Table 1, Figure 2). Whereas, city of Jinhua, Quzhou, and Lishui cities had the highest hospitalization rates with 17.22, 16.17, and 14.13%, respectively.

### 3.2. Trend and seasonality

**Figure 3:**
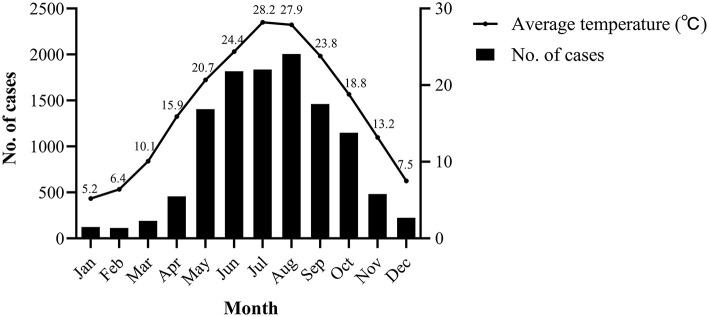
Temporal distribution and number of *Salmonella* cases, by month of occurrence, 2012–2021.

In terms of temporal distribution, *Salmonella* infection mainly occurred seasonally from May to October, during which 8,754 cases occurred, accounting for 77.69% of the total cases. These months are the hottest in Zhejiang, with average temperature ranging between 20.7 and 28.2°C (Figure 3) (23). Moreover, the highest positive rate (5.21%) was observed in summer (June to August) (*P* < 0.001) (Table 1).

### 3.3. Age, gender, and occupational difference

The average age of 11,269 patients (6,155 males and 5,114 females) was 33.63 years. A slight difference was observed between the different sex groups (*P* = 0.012), as shown in Table 1. As for age distribution, the majority of reported *Salmonella* cases affected children aged 0–4 years (4,060 cases, 36.02%), and older adults aged >60 years (2,109 cases, 18.72%), with positive rates of 8.79 and 4.34%, and hospitalization rates of 48.14 and 27.70%, respectively. A significant occupational difference was observed between the occupational groups (*P* < 0.001). The positive infection rate was the highest in scattered children (3,136 cases, 13.02%), with a hospitalization rate of 37.73%.

### 3.4. Implicated foods and settings

Among the 11,269 *Salmonella* cases, 1,434 (12.73%) were attributed to fruits and fruit products (Table 2). Aquatic products (1,370 cases, 12.16%), meat and meat products (1,337 cases, 11.86%), cereals and grain products (1,054 cases, 9.35%), milk and dairy products (705 cases, 6.26%), vegetables and vegetable products (657 cases, 5.83%), eggs and egg products (493 cases, 4.37%), beverages and frozen drinks (273 cases, 2.42%), infant foods (226 cases, 2.01%), and beans and soy products (186 cases, 1.65%), these were the most commonly reported food items. Approximately 6.85% (772/11,269) of the cases were associated with mixed dishes, 6.05% (682/11,269) with multiple foods, and 12.66% (1,427/11,269) with unknown food. In addition, 653 (5.79%) cases were relevant to other food products containing liquor products, fungi, nuts, sweets, and water. Among single food category, fruit and fruit products (186 hospitalizations, 11.52%) were responsible for most hospitalizations, followed by meat and meat products (168 hospitalizations, 10.41%) and cereals and grain products (156 hospitalizations, 9.67%).

**Table 2:** Food and Setting distribution of *Salmonella* positive cases in Zhejiang Province from 2012 to 2021.

**Variables**	**Case**		**Hospitalizations**	
	***n***	**%**	***n***	**%**
**Food**				
Fruits and fruit products	1,434	12.73	186	11.52
Aquatic products	1,370	12.16	126	7.81
Meat and meat products	1,337	11.86	168	10.41
Cereals and grain products	1,054	9.35	156	9.67
Milk and dairy products	705	6.26	118	7.31
Vegetables and vegetable products	657	5.83	86	5.33
Eggs and egg products	493	4.37	80	4.96
Beverages and frozen drinks	273	2.42	18	1.12
Infant foods	226	2.01	36	2.23
Beans and soy products	186	1.65	26	1.61
Mixed dishes	772	6.85	97	6.01
Multiple foods	682	6.05	124	7.68
Unknown	1,427	12.66	282	17.47
Others	653	5.79	111	6.88
**Setting**				
Household	6,163	54.69	772	47.83
Restaurant	479	4.25	33	2.04
Retail	182	1.62	36	2.23
Collective canteens	142	1.26	7	0.43
School	30	0.27	8	0.50
Rural banquet	28	0.25	2	0.12
Unknown	1,525	13.53	309	19.14
Others	2,720	24.14	490	30.36

The distribution of cases according to the setting is shown in Table 2. *Salmonella* FBDs occurred most frequently in household settings (6,163 cases, 54.69%), followed by restaurants (479 cases, 4.25%), retail (182 cases, 1.61%), collective canteen (142 cases, 1.26%), schools (30 cases, 0.27%), rural banquets (28, 0.25%), and other settings (4,245 cases, 37.67%), including unknown settings (1,312 cases, chophouses, street stalls, and delivering meals). *Salmonella* FBDs in households (772 hospitalizations, 47.83%), retail (36 hospitalizations, 2.23%), and restaurants (33 hospitalizations, 2.04%) resulted in a relatively high numbers of hospitalizations.

### 3.5. Serotypes and symptoms

In this study, 173 *Salmonella* serotypes were identified. *Salmonella typhimurium* was the most common serotype, accounting for 36.07% (4,065/11,269), and *Salmonella enteritidis* was the second, accounting for 15.17% (1,710/11,269), followed by *Salmonella london*, accounting for 6.05% (682/11,269). Among the 11,269 *Salmonella* cases, 99.41% had diarrhea, 47.22% had abdominal pain, 27.03% had fever, 20.05% had nausea, and 18.46% had vomiting. Symptoms varied greatly according to serotype. Diarrhea was the most common symptom among the serotypes (Table 3).

**Table 3:** Reported signs and symptoms of *Salmonella* cases in different serotypes.

**Variables**	**Typhimurium** **(*N* = 4,065)**	**Enteritidis** **(*N* = 1,710)**	**London** **(*N* = 682)**	**Derby** **(*N* = 210)**	**Risson** **(*N* = 149)**	**Stanley** **(*N* = 146)**	**Dublin** **(*N* = 145)**	**Gold Coast** **(*N* = 137)**	**Paratyphi** **(*N* = 134)**	**Rosenticus** **(*N* = 128)**	**Infantis** **(*N* = 124)**	**Sick cattle** **(*N* = 117)**
Diarrhea	4,012 (98.70)	1,688 (98.71)	674 (98.83)	210 (100.00)	146 (97.99)	145 (99.32)	145 (100.00)	136 (99.27)	133 (99.25)	126 (98.44)	123 (99.19)	111 (94.87)
Abdominal pain	1,784 (43.89)	944 (55.20)	381 (55.87)	82 (39.05)	76 (51.01)	57 (39.04)	88 (60.69)	81 (59.12)	64 (47.76)	54 (42.19)	56 (45.16)	57 (48.72)
Fever (≥37.5°C)	1,204 (29.62)	458 (26.78)	136 (19.94)	38 (18.10)	36 (24.16)	37 (25.34)	67 (46.21)	31 (22.63)	21 (15.67)	17 (13.28)	34 (27.42)	21 (17.95)
Vomiting	688 (16.92)	408 (23.86)	116 (17.01)	42 (20.00)	22 (14.77)	28 (19.18)	48 (33.10)	19 (13.87)	22 (16.42)	24 (18.75)	19 (15.32)	17 (14.53)
Nausea	675 (16.61)	427 (24.97)	156 (22.87)	46 (21.90)	33 (22.15)	27 (18.49)	42 (28.97)	27 (19.71)	21 (15.67)	26 (20.31)	25 (20.16)	16 (13.68)
Debilitation	269 (6.62)	157 (9.18)	55 (8.06)	16 (7.62)	14 (9.40)	12 (8.22)	11 (7.59)	11 (8.03)	13 (9.70)	7 (5.47)	13 (10.48)	7 (5.98)
Thirsty	115 (2.83)	77 (4.50)	20 (2.93)	6 (2.86)	5 (3.36)	7 (4.79)	3 (2.07)	4 (2.92)	8 (5.97)	3 (2.34)	3 (2.42)	5 (4.27)
Hypourocrinia	113 (2.78)	51 (2.98)	22 (3.23)	8 (3.81)	2 (1.34)	4 (2.74)	6 (4.14)	4 (2.92)	7 (5.22)	1 (0.78)	3 (2.42)	4 (3.42)
Dehydration	94 (2.31)	26 (1.52)	11 (1.61)	3 (1.43)	2 (1.34)	5 (3.42)	2 (1.38)	2 (1.46)	2 (1.49)	1 (0.78)	3 (2.42)	1 (0.85)
Tenesmus	51 (1.25)	17 (0.99)	6 (0.88)	2 (0.95)	1 (0.67)	3 (2.05)	3 (2.07)	3 (2.19)	1 (0.75)	2 (1.56)	4 (3.23)	0 (0.00)
Shiver	28 (0.69)	15 (0.88)	5 (0.73)	0 (0.00)	6 (4.03)	3 (2.05)	2 (1.38)	0 (0.00)	0 (0.00)	1 (0.78)	1 (0.81)	2 (1.71)
Flushed face	26 (0.64)	16 (0.94)	3 (0.44)	1 (0.48)	1 (0.67)	2 (1.37)	0 (0.00)	0 (0.00)	0 (0.00)	0 (0.00)	0 (0.00)	1 (0.85)
Pale	25 (0.62)	8 (0.47)	2 (0.29)	3 (1.43)	1 (0.67)	2 (1.37)	0 (0.00)	0 (0.00)	0 (0.00)	0 (0.00)	0 (0.00)	0 (0.00)
Headache	18 (0.44)	17 (0.99)	4 (0.59)	1 (0.48)	0 (0.00)	0 (0.00)	2 (1.38)	0 (0.00)	0 (0.00)	1 (0.78)	2 (1.61)	0 (0.00)
Weight loss	15 (0.37)	6 (0.35)	2 (0.29)	1 (0.48)	0 (0.00)	0 (0.00)	0 (0.00)	0 (0.00)	0 (0.00)	0 (0.00)	0 (0.00)	0 (0.00)

## 4. Discussion

*Salmonella* infection is a vital public health concern in the Zhejiang Province. In this study, we for the first time described the epidemiological and etiological characteristics of the *Salmonella* infection in the Zhejiang Province between 2012 and 2021. During the years, 11,269 cases with 1,614 (14.32%) hospitalizations were reported, corresponding to an average positive rate of 3.65% for the whole province. The average age of patients infected with *Salmonella* was 33.63 years. In all settings and food categories, *Salmonella* cases occurred most commonly in household settings (6,163 cases, 54.69%) due to fruit and fruit products (1,434 cases, 12.73%).

The positive rate of *Salmonella* infection increased during 2012–2021 and remained particularly high between 2020 and 2021. Considering the gradual improvement of the surveillance system at all levels of CDCs and hospitals, more attention has been paid to FBDs and cases have been reported in detail (24). Compared to the rates abroad, the CDC estimated that *Salmonella enterica* caused 1.2 million infections, 24,000 hospitalizations, and 450 deaths in the United States (25). According to the European Food Safety Authority (EFSA) and European CDC (ECDC) reports, 88,715 confirmed cases of *Salmonella* infection and an EU notification rate of 23.4 cases per 100,000 population were recorded (26). South East Asia, with 11 different countries, ranks third as the super region for the global burden of *Salmonella*-induced gastroenteritis (27). Some epidemiological studies have revealed the prevalence, characterization, genetic investigation, serovar distribution, and antibiotic resistance in China, however, the results remain ambiguous (28–30). Therefore, it is reasonable to assume that FBD caused due to *Salmonella* infection is a growing public health issue in the Zhejiang Province.

*Salmonella* infection showed an obvious pattern according to age, and young children and older adults were especially vulnerable. Following are some plausible explanations as to why young children and older adults are more susceptible to *Salmonella* infection. Primarily, immunocompromised children and older adults and those with underlying conditions are particularly vulnerable to invasive diseases (31, 32). Due to their immature immune systems and permeable gastrointestinal tracts, infants and young children are more susceptible to infection by foodborne pathogenic bacteria than other age groups (33). Older adults exhibit dysregulated immune responses to pathogens. In addition, consumption of infant formula contaminated with *Salmonella* may result in serious illness. In terms of community risks, powdered infant formula contamination and its associated hazards may not be fully recognized (34, 35). Moreover, parents pay high attention to *Salmonella* infection, and they tend to seek medical advice (36).

Regional differences in the distribution of *Salmonella* were observed in the present study. Considering the location of the Zhejiang Province, the annual mean temperature ranges from 15.0 to 18.0°C and the province experience a subtropical humid climate. Taizhou was deemed to have the highest rate of *Salmonella* positivity, with a population of more than six million and 73.0% mountainous area. Hangzhou, with the most cases of *Salmonella* infection, has the lowest percentage of hospitalizations. To the best of our knowledge, in many locations with limited resources, food safety methods for prevention are rarely the main focus, and the lack of food safety knowledge is a vital reason for FBDs (37). With the diverse species of *Salmonella* serotypes, there are differences in the biofilm lifestyles, long-term persistence outside, and immune responses (38). Recent studies have revealed that climate and seasonality may play important roles in the prevalence of *Salmonella* (39). In this study, *Salmonella* infection showed a significantly increased positive rates in the warm seasons, especially in summer. Stronger research evidence indicated that *Salmonella* infections are elevated in warm climates (40, 41). Owing to high temperatures, people prefer raw and cold foods. Frozen, raw, and cold foods, such as meat, milk and milk products, have been identified as risk factors for *Salmonella* infection (42). Furthermore, warm and suitable temperatures are more suitable for the growth of *Salmonella*. Therefore, refrigerating foods is necessary for the prevention and control of bacterial FBDs.

Interestingly, more than half of the *Salmonella* positive cases occurred in household setting (6,163 case, 54.69%) in our study. According to a survey of six European countries, approximately 40% of foodborne infections are acquired at home because of cross-contamination and food preferences (43). The food category results demonstrated that fruits and fruit products (1,434 cases, 12.73%), aquatic products (1,370 cases, 12.16%), and meat and meat products (1,337 cases, 11.86%) acquired the top three positions among all food categories in Zhejiang Province that caused *Salmonella* infections. The main sources of *Salmonella* infection in humans are meat products, including the consumption of contaminated poultry meat at the global level (44). A systematic review and meta-analysis had evaluated that the prevalence level differed from high to low among raw poultry meat, including chicken, pigeon, duck, and other poultry meat (9). However, an increasing number of reports have linked *Salmonella* contaminated raw vegetables and fruits with food poisoning (45). *Salmonella* uses multiple strategies to manipulate the host defense system while in contact with fruits and vegetables, including affecting the genetic variation, controlling the heterogenous expression of flagellin, and suppressing the dual expression of effector proteins (46).

Serotyping results demonstrated that most *Salmonella* FBDs were caused by multiple serotypes, including *S. typhimurium, S. enteritidis*, and *S. london*, which is consistent with previous studies (47, 48). In the past two decades, *S. typhimurium* and *S. enteritidis* have become the most common *Salmonella* serotypes responsible for human infections in different regions (49). An epidemiological investigation showed that high levels of *Salmonella* contamination were detected in meat products, and multiple virulence-associated genes were isolated in Southern China, Guangdong Province (50). Some valuable baseline data collected from other provincial regions also showed significant differences in *Salmonella* serotypes (51, 52). It is noteworthy that *S. derby, S. risoson, S. stanley, S. dublin, S. gold coast, S. paratyphi, S. rosenticus, S. infantis*, and *S. sick cattle* were also considerable serovars, accounting for 1.86, 1.32, 1.30, 1.29, 1.22, 1.19, 1.14, 1.10, and 1.04% of the total serotypes, respectively. Moreover, *S. stanley, S. dublin*, and *S. sick bovine* were higher in the city of Jinhua, Quzhou, and Zhoushan, respectively, which indicated that there were some differences in the distribution of the dominant serotypes. These differences may be associated with geographical location, eating habits, climatic conditions, and food preferences. Therefore, it is essential to systematically monitor the *Salmonella* serotype distribution through a proper sampling layout.

In terms of clinical symptoms, our results showed that most serotypes could cause AGI symptoms, including diarrhea, abdominal pain, fever, nausea, and vomiting. The twelve main serotypes caused diarrhea in more than 90% of cases. The proportion of abdominal pain was the highest in *S. dublin* (60.69%) and lowest in *S. stanley* (39.04%). Remarkably, *S. dublin* (46.21%) was responsible for the highest fever proportion, whereas it was lowest in *S. rosenticus* (13.28%). Nevertheless, fever caused by *Salmonella* infection may be difficult to distinguish from other febrile diseases; therefore, etiological examination is essential (18). The highest proportions of nausea and vomiting were caused by S. *dublin*. *S. dublin* mainly colonizes cattle; however, upon infection, it might lead to invasive illness in humans (53).

This study had some limitations. First of all, the case data were collected through the NFDSN, which is a passive surveillance system and some information was either missing or incomplete, such as food categories, settings and etc., so the conclusions might not be representative of unknown classification. Second, although our surveillance system has improved significantly since 2012 in all province, the data quality is still related to regional distribution, local economic level, detection capacity, and coordination degree. Additionally, food information was self-reported by patients, so there was great uncertainty regarding epidemiological tracing. Further case surveillance should focus on the etiology and food, and also training investigators to make efforts to obtain the exact causes of FBDs and accurate characteristics.

## 5. Conclusion

We have demonstrated for the first time of epidemiological characteristics for foodborne diseases caused by *Salmonella* in China over the past 10 years. Since *Salmonella* infections continues to be a severe public health concern worldwide, we recommend that the data accuracy of food collection for suspected exposure should be optimized and compared with the actual contamination results in food items to provide support for supervision. To prevent and control future FBDs caused by *Salmonella*, it is necessary to carry out drug resistance analysis and whole genome sequencing of *Salmonella* cases, and further explore its biological mechanism. There is a need to carry out an overall assessment of *Salmonella* infection in residents by strengthening FBDs surveillance, source attribution and burden estimation, and more efforts should be directed toward conducting comprehensive assessments for specific public health policy formulation.

## Data availability statement

The datasets presented in this article are not readily available because Ethics Committee of Zhejiang Provincial Center for Disease Control and Prevention. Requests to access the datasets should be directed to xjqi@cdc.zj.cn.

## Ethics statement

The studies involving human participants were reviewed and approved by Ethics Committee of Zhejiang Provincial Center for Disease Control and Prevention. Written informed consent from the participants' legal guardian/next of kin was not required to participate in this study in accordance with the national legislation and the institutional requirements.

## Author contributions

Conceptualization and writing—review and editing: YH and XQ. Data curation: JW. Investigation: LC. Methodology: HZ and YH. Project administration: XQ and JC. Supervision: JC. Validation: RZ. Writing—original draft: YH. All authors have read and agreed to the published version of the manuscript.

## References

[1] Faour-Klingbeil D E CDT. Prevention and control of foodborne diseases in Middle-East North African countries: review of national control systems. Int J Environ Res Public Health. (2019) 17:70. 10.3390/ijerph17010070 31861843 PMC6982137

[2] Torgerson PR Devleesschauwer B Praet N Speybroeck N Willingham AL Kasuga F. World health organization estimates of the global and regional disease burden of 11 foodborne parasitic diseases, 2010: a data synthesis. PLoS Med. (2015) 12:e1001920. 10.1371/journal.pmed.1001920 26633705 PMC4668834

[3] Gallo M Ferrara L Calogero A Montesano D Naviglio D. Relationships between food and diseases: what to know to ensure food safety. Food Res Int. (2020) 137:109414. 10.1016/j.foodres.2020.109414 33233102

[4] Martinović T Andjelković U Gajdošik M Rešetar D Josić D. Foodborne pathogens and their toxins. J Proteomics. (2016) 147:226–35. 10.1016/j.jprot.2016.04.029 27109345

[5] Pucciarelli MG García-Del Portillo F. *Salmonella* intracellular lifestyles and their impact on host-to-host transmission. Microbiol Spectrum. (2017) 5:2016. 10.1128/microbiolspec.MTBP-0009-2016 28730976 PMC11687531

[6] Dutta D Kaushik A Kumar D Bag S. Foodborne pathogenic vibrios: antimicrobial resistance. Front Microbiol. (2021) 12:638331. 10.3389/fmicb.2021.638331 34276582 PMC8278402

[7] Jordan K McAuliffe O. Listeria monocytogenes in foods. Adv Food Nutr Res. (2018) 86:181–213. 10.1016/bs.afnr.2018.02.006 30077222

[8] Kadariya J Smith TC Thapaliya D. Staphylococcus aureus and staphylococcal food-borne disease: an ongoing challenge in public health. Biomed Res Int. (2014) 2014:827965. 10.1155/2014/827965 24804250 PMC3988705

[9] Sun T Liu Y Qin X Aspridou Z Zheng J Wang X. The prevalence and epidemiology of *Salmonella* in retail raw poultry meat in china: a systematic review and meta-analysis. Foods. (2021) 10:2757. 10.3390/foods10112757 34829037 PMC8622452

[10] Whiley H Ross K. *Salmonella* and eggs: from production to plate. Int J Environ Res Public Health. (2015) 12:2543–56. 10.3390/ijerph120302543 25730295 PMC4377917

[11] Chlebicz A Slizewska K. *Campylobacteriosis, Salmonellosis, Yersiniosis*, and *Listeriosis* as Zoonotic foodborne diseases: a review. Int J Environ Res Public Health. (2018) 15:863. 10.3390/ijerph15050863 29701663 PMC5981902

[12] Castro-Vargas RE Herrera-Sánchez MP Rodríguez-Hernández R Rondón-Barragán IS. Antibiotic resistance in *Salmonella* spp. Isolated from poultry: a global overview. Vet world. (2020) 13:2070–84. 10.14202/vetworld.2020.2070-2084 33281339 PMC7704309

[13] Hung YT Lay CJ Wang CL Koo M. Characteristics of non-typhoidal *Salmonella*g astroenteritis in Taiwanese children: a 9-year period retrospective medical record review. J Infect Public Health. (2017) 10:518–21. 10.1016/j.jiph.2016.09.018 28209468

[14] Yeh Y Purushothaman P Gupta N Ragnone M Verma SC de Mello AS. Bacteriophage application on red meats and poultry: effects on *Salmonella* population in final ground products. Meat Sci. (2017) 127:30–4. 10.1016/j.meatsci.2017.01.001 28110127

[15] Wessels K Rip D Gouws P. *Salmonella* in chicken meat: consumption, outbreaks, characteristics, current control methods and the potential of bacteriophage use. Foods. (2021) 10:1742. 10.3390/foods10081742 34441520 PMC8394320

[16] Kore K Asrade B Demissie K Aragaw K. Characterization of *Salmonella* isolated from apparently healthy slaughtered cattle and retail beef in Hawassa, Southern Ethiopia. Prev Vet Med. (2017) 147:11–6. 10.1016/j.prevetmed.2017.08.018 29254708

[17] Milczarek M Sadkowska-Todys M Czarkowski MP Kitowska W. *Salmonellosis* in Poland in 2017. Przegl Epidemiol. (2019) 73:463–77. 10.32394/pe.73.44 32237696

[18] Crump JA Sjölund-Karlsson M Gordon MA Parry CM. Epidemiology, clinical presentation, laboratory diagnosis, antimicrobial resistance, and antimicrobial management of invasive *Salmonella* infections. Clin Microbiol Rev. (2015) 28:901–37. 10.1128/CMR.00002-15 26180063 PMC4503790

[19] Powell MR Crim SM Hoekstra RM Williams MS Gu W. Temporal patterns in principal *Salmonella* serotypes in the USA 1996–2014. Epidemiol Infect. (2018) 146:437–41. 10.1017/S0950268818000195 29436316 PMC9134518

[20] Ford L Miller M Cawthorne A Fearnley E Kirk M. Approaches to the surveillance of foodborne disease: a review of the evidence. Foodborne Pathog Dis. (2015) 12:927–36. 10.1089/fpd.2015.2013 26554434

[21] Chen L Sun L Zhang R Liao N Qi X Chen J. Surveillance for foodborne disease outbreaks in Zhejiang Province, China, 2015–2020. BMC Public Health. (2022) 22:135. 10.1186/s12889-022-12568-4 35045858 PMC8769373

[22] Zhejiang Provincial Bureau of Statistics. Available online at: http://tjj.zj.gov.cn/ (accessed December 15, 2022).

[23] Sun L Zhang H Chen J Chen L Qi X Zhang R. Epidemiology of foodborne disease outbreaks caused by non-typhoidal *Salmonella* in Zhejiang Province, China, 2010–2019. Foodborne Pathog Dis. (2021) 18:880–6. 10.1089/fpd.2021.0006 34357797

[24] Chen L Wang J Zhang R Zhang H Qi X He Y. An 11-year analysis of bacterial foodborne disease outbreaks in Zhejiang Province, China. Foods. (2022) 11:2382. 10.3390/foods11162382 36010382 PMC9407109

[25] Harvey RR Friedman CR Crim SM Judd M Barrett KA Tolar B. Epidemiology of *Salmonella* enterica Serotype Dublin Infections among Humans, United States, 1968–2013. Emerg Infect Dis. (2017) 23:1493–501. 10.3201/eid2309.170136 28820133 PMC5572876

[26] Bonardi S. *Salmonella* in the pork production chain and its impact on human health in the European Union. Epidemiol Infect. (2017) 145:1513–26. 10.1017/S095026881700036X 28241896 PMC9203350

[27] Patra SD Mohakud NK Panda RK Sahu BR Suar M. Prevalence and multidrug resistance in *Salmonella* enterica Typhimurium: an overview in South East Asia. World J Microbiol Biotechnol. (2021) 37:185. 10.1007/s11274-021-03146-8 34580741

[28] Shen W Chen H Geng J Wu RA Wang X Ding T. Prevalence, serovar distribution, and antibiotic resistance of *Salmonella* spp. isolated from pork in China: a systematic review and meta-analysis. Int J Food Microbiol. (2022) 361:109473. 10.1016/j.ijfoodmicro.2021.109473 34768041

[29] Zhao X Hu M Zhang Q Zhao C Zhang Y Li L. Characterization of integrons and antimicrobial resistance in *Salmonella* from broilers in Shandong, China. Poult Sci. (2020) 99:7046–54. 10.1016/j.psj.2020.09.071 33248621 PMC7705031

[30] Jiu Y Meng X Hong X Huang Q Wang C Chen Z. Prevalence and characterization of *Salmonella* in three typical commercial pig abattoirs in Wuhan, China. Foodborne Pathog Dis. (2020) 17:620–7. 10.1089/fpd.2019.2737 32130028

[31] Wen SC Best E Nourse C. Non-typhoidal *Salmonella* infections in children: review of literature and recommendations for management. J Paediatr Child Health. (2017) 53:936–41. 10.1111/jpc.13585 28556448

[32] Allen JC Toapanta FR Chen W Tennant SM. Understanding immunosenescence and its impact on vaccination of older adults. Vaccine. (2020) 38:8264–72. 10.1016/j.vaccine.2020.11.002 33229108 PMC7719605

[33] Kent RM Fitzgerald GF Hill C Stanton C Ross RP. Novel approaches to improve the intrinsic microbiological safety of powdered infant milk formula. Nutrients. (2015) 7:1217–44. 10.3390/nu7021217 25685987 PMC4344585

[34] Blackshaw K Valtchev P Koolaji N Berry N Schindeler A Dehghani F. The risk of infectious pathogens in breast-feeding, donated human milk and breast milk substitutes. Public Health Nutr. (2021) 24:1725–40. 10.1017/S1368980020000555 32539885 PMC10195434

[35] Yang B Zhao H Cui S Wang Y Xia X Xi M. Prevalence and characterization of *Salmonella* enterica in dried milk-related infant foods in Shaanxi, China. J Dairy Sci. (2014) 97:6754–60. 10.3168/jds.2014-8292 25218754

[36] Behera JR Rup AR Dash AK Sahu SK Gaurav A Gupta A. Clinical and laboratory profile of enteric fever in children from a tertiary care centre in Odisha, Eastern India. Cureus. (2021) 13:e12826. 10.7759/cureus.12826 33633872 PMC7899128

[37] Chapman B Gunter C. Local food systems food safety concerns. Microbiol Spect. (2018) 6:2017. 10.1128/microbiolspec.PFS-0020-2017 29651980 PMC11633565

[38] Harrell JE Hahn MM D'Souza SJ Vasicek EM Sandala JL Gunn JS. *Salmonella* biofilm formation, chronic infection, and immunity within the intestine and hepatobiliary tract. Front Cell Infect Microbiol. (2020) 10:624622. 10.3389/fcimb.2020.624622 33604308 PMC7885405

[39] Smith BA Meadows S Meyers R Parmley EJ Fazil A. Seasonality and zoonotic foodborne pathogens in Canada: relationships between climate and *Campylobacter, E. coli*, and *Salmonella* in meat products. Epidemiol Infect. (2019) 147:e190. 10.1017/S0950268819000797 31364535 PMC6518574

[40] Lake IR. Food-borne disease and climate change in the United Kingdom. Environ Health Glob Access Sci Sou. (2017) 16:117. 10.1186/s12940-017-0327-0 29219100 PMC5773878

[41] Naumova EN Jagai JS Matyas B DeMaria A Jr MacNeill IB Griffiths JK. Seasonality in six enterically transmitted diseases and ambient temperature. Epidemiol Infect. (2007) 135:281–92. 10.1017/S0950268806006698 17291363 PMC2870561

[42] Morton VK Kearney A Coleman S Viswanathan M Chau K Orr A. Outbreaks of *Salmonella* illness associated with frozen raw breaded chicken products in Canada, 2015–2019. Epidemiol Infect. (2019) 147:e254. 10.1017/S0950268819001432 31436145 PMC6805751

[43] Møretrø T Nguyen-The C Didier P Maître I Izsó T Kasza G. Consumer practices and prevalence of *Campylobacter, Salmonella*, and norovirus in kitchens from six European countries. Int J Food Microbiol. (2021) 347:109172. 10.1016/j.ijfoodmicro.2021.109172 33812164

[44] Antunes P Mourão J Campos J Peixe L. *Salmonellosis*: the role of poultry meat. Clinical microbiology and infection. Eur Soc Clin Microbiol Infect Dis. (2016) 22:110–21. 10.1016/j.cmi.2015.12.004 26708671

[45] Wiedemann A Virlogeux-Payant I Chaussé AM Schikora A Velge P. Interactions of *Salmonella* with animals and plants. Front Microbiol. (2014) 5:791. 10.3389/fmicb.2014.00791 25653644 PMC4301013

[46] Zarkani AA Schikora A. Mechanisms adopted by *Salmonella* to colonize plant hosts. Food Microbiol. (2021) 99:103833. 10.1016/j.fm.2021.103833 34119117

[47] Xu H Zhang W Zhang K Zhang Y Wang Z Zhang W. Characterization of *Salmonella* serotypes prevalent in asymptomatic people and patients. BMC Infect Dis. (2021) 21:632. 10.1186/s12879-021-06340-z 34210275 PMC8252320

[48] Kim SH Sung GH Park EH Hwang IY Kim GR Song SA. Serotype Distribution and Antimicrobial Resistance of *Salmonella* Isolates in Korea between 2016 and 2017. Ann Lab Med. (2022) 42:268–73. 10.3343/alm.2022.42.2.268 34635618 PMC8548255

[49] Sun H Wan Y Du P Bai L. The epidemiology of monophasic *Salmonella* typhimurium. Foodborne Pathog Dis. (2020) 17:87–97. 10.1089/fpd.2019.2676 31532231

[50] Chen Z Bai J Wang S Zhang X Zhan Z Shen H. Prevalence, antimicrobial resistance, virulence genes and genetic diversity of *Salmonella* Isolated from retail duck meat in Southern China. Microorganisms. (2020) 8:444. 10.3390/microorganisms8030444 32245148 PMC7143943

[51] Li Y Yang X Zhang H Jia H Liu X Yu B. Prevalence and antimicrobial susceptibility of *Salmonella* in the commercial eggs in China. Int J Food Microbiol. (2020) 325:108623. 10.1016/j.ijfoodmicro.2020.108623 32339770

[52] Zheng D Ma K Du J Zhou Y Wu G Qiao X. Characterization of human origin *Salmonella* Serovar 1,4,[5],12:i:- in Eastern China, 2014 to 2018. Foodborne Pathog Dis. (2021) 18:790–7. 10.1089/fpd.2021.0008 34287022

[53] Betancor L Yim L Martínez A Fookes M Sasias S Schelotto F. Genomic comparison of the closely related *Salmonella* enterica *Serovars Enteritidis* and Dublin. Open Microbiol J. (2012) 6:5–13. 10.2174/1874285801206010005 22371816 PMC3282883

